# Effects of a Coordinative Ability Training Program on Adolescents’ Cognitive Functioning

**DOI:** 10.3389/fpsyg.2021.620440

**Published:** 2021-01-28

**Authors:** Francesca Latino, Stefania Cataldi, Francesco Fischetti

**Affiliations:** Department of Basic Medical Sciences, Neurosciences and Sense Organs, School of Medicine, University of Bari, Bari, Italy

**Keywords:** physical education, Corsi’s block-tapping test, cognitive performance, academic achievement, exercise

## Abstract

The purpose of this randomized controlled study was to investigate the effects of a 12-week coordinative ability training program on adolescents’ cognitive functioning, using evaluation tests of visuospatial perception, attention, and working memory. We randomly assigned 60 public school students (14–15 years) to either an experimental coordinative abilities training (∼40 min twice/week) group (*n* = 30) or a control group (*n* = 30) who received general psycho-physical wellness training (∼40 min., twice a week). At baseline and after training we used two standardized motor tests and a single cognitive measure (Corsi’s Block-tapping test) to assess students’ visuospatial perception, attention, and working memory. We found a significant Time x Group interaction for the Throwing and Catching Test and Corsi’s Block-Tapping test, reflecting a meaningful experimental group improvement (*p* < 0.001), and there were no significant pre-post changes found in the control group. Thus, a 12-week program of coordinative abilities was able to improve not only coordination skills but aspects of cognitive functioning relevant to academic achievement.

## Introduction

Physical exercise represents a natural and productive opportunity for both physical and cognitive development. Exercise can help people feel better about themselves, decreases risk of diseases and improves learning (Janssen and LeBlanc, 2010).

This occurs because a regular engagement in physical exercise produces numerous health benefits in the brain, by inducing structural and functional changes (Singh et al., 2019). First of all, it increases gray matter volume in frontal and hippocampal regions, increases blood flow, and affects brain plasticity (Fernandes et al., 2017). Moreover, while exercising, the organism releases several neurotrophic molecules, such as peripheral brain-derived neurotrophic factor (BDNF), that stimulate hippocampal neurogenesis, brain angiogenesis, and the synthesis of monoamines (Sibley and Etnier, 2003; Castelli et al., 2014; Donnelly et al., 2016; Vazou et al., 2016; Stimpson et al., 2018; Singh et al., 2019). Thus, the constant practice of physical activity leads to durable changes enhancing brain integrity and brain functioning and, consequently, cognitive health. These effects are reflected on cognitive functioning and consequently on academic achievement (Hötting and Röder, 2013).

Several studies have indicated that children and adolescent who spend more time in physical activities have better academic performance than those who are sedentary (Ayers and Sariscsany, 2010). Researchers suggesting that young people may improve mental acuity, skills, and strategies through physical exercise (Donnelly et al., 2016) and supports the idea that cognitive performance improves with physical exercise that makes adolescents more efficient on reaction time tasks and more flexible on attention-orientation tasks (Muiños and Ballesteros, 2014, 2018; Alesi et al., 2016; Zach and Shalom, 2016; Burns et al., 2017; Kashfi et al., 2019). Furthermore, a well-managed physical exercise may facilitate certain aspects of information processing in children and adolescent (Tomporowski et al., 2011).

Recent findings also highlight a significant role for coordinative exercise in improving academic performance (Kwok et al., 2011; Hotting et al., 2012). Cognitive performance seems to be influenced by bilateral coordinative exercise that shows benefits even after short bouts of exercise (Budde et al., 2008), particularly on tasks that involve executive function (Yu-Kai et al., 2013). Coordinative exercise of both low and moderate intensities may also increase visuospatial perception, attentional resources, working memory and shorten the time needed for neurocognitive processing (Yu-Kai et al., 2013). These might be explained by the fact that the coordinative component of the movement increases synapses in important brain areas such as the cerebellum (Donnelly et al., 2016). Complex movement patterns engage the cerebellum which affects areas such as attention and memory, functions that are affected by cerebellum (Guillamón et al., 2020). Specifically, visuospatial attention is important in the performance of a variety of activities and plays a central role for processing visual information and perceiving moving stimuli (Guo et al., 2016). A series of studies support the idea that physical exercise make children and adolescent more efficient than sedentary, in attention-orientation tasks (Guillamón et al., 2020). Similarly, working memory, that is the ability to maintain and consciously manipulate information, is particularly age-sensitive (Baddeley et al., 1999; Kirova et al., 2015) and seems to be particularly flexible at a younger ages. Several research have indicated that it may benefit from coordinative exercise (Chang et al., 2013; Padilla et al., 2014) and reacts positively to environmental changes (Carretti et al., 2007; Blacker et al., 2014).

In light of the above, cognitive benefits of coordinative exercise may have important implications for improving students’ academic performance and preventing academic failure. Therefore, to extend the understanding of these connections, the main goal of this study was to investigate the association between coordinative physical exercise and cognition, based on the assumption that coordinative exercise could improve adolescents’ visuospatial perception (such as hand-eye coordination and motion spatial planning), attention and working memory, important functions for learning.

## Materials and Methods

### Study Design

We employed a randomized controlled study design to investigate the effects of a coordinative ability training program vs. a control condition of psycho-physical well-being training on students’ visuospatial, attentive and memory skills. The study was high school-based, with the intervention involving 24 lessons over 12 weeks and student assessment occurring at the 1st and 12th weeks. Thus, we collected and recorded data at baseline (pre-test) and after 12 weeks (post-test). After pre-test, we randomly assigned participants to the experimental group involving a coordinative abilities training protocol designed to improve cognitive skills or a control group in which students received group training for general psycho-physical wellness. To allow statistically meaningful comparisons between different type of activities, students were classified as participants in activities that shared similar characteristics.

### Participants

We recruited 60 healthy male and female adolescent students, aged 14–15 years (30 males and 30 females; *M* age = 14.4, *SD* = 0.5 years*; M* height = 167.5, *SD* = 7.4 cm; *M* weight = 60.1, *SD* = 9.39 kg; *M* BMI = 21.36, *SD* = 2.0 kg), to participate in the study. Participants were from the same socio-economic background and at the time of data collection, all were attending their first year of high school. We conducted an *a priori* statistical power calculation to determine the sample size required to detect changes in the dependent measures resulting from the intervention (Faul et al., 2007). We assumed a type I error of 0.05 and a type II error rate of 0.10 (90% statistical power), and we assumed an effect size of 0.25. We used a statistical software application called G-Power 3.1 and found that 46 participants in total would be sufficient to observe Time × Group interaction effects in analysis of variance.

We matched participants based on their gender and randomly allocated them into the experimental group (*n* = 30) or control group (*n* = 30). Inclusion criteria were the following: participants had to be relatively healthy individuals capable of completing a moderate-intensity aerobic exercise session and able to abstain from all physical activity outside the parameters of the study protocol. Any student older than 15 years of age, with an individual education plan as a result of a disability or who had an orthopedic condition that would limit their ability to perform exercise, were excluded from the study. The study was conducted from March to May 2019. All participants and their parents received a complete explanation in advance about the purpose of the experiment, its contents, and safety issues based on the Declaration of Helsinki, and parents of all participants provided their written informed consent before the study.

### Procedures

We administered the intervention program at a local public school during normal school days. Both the experimental and control group met twice per week for 60 min each day (approximately 40 min of activity time), under carefully monitored and controlled conditions. Each session of the intervention program involved the following stages: warm-up for 10 min, perform the main exercises for 25 min, and cool down for 5 min. Specifically, warm-up consisted of low to moderate intensity aerobic exercise, while the exercise phase was designed to reach and emphasize different coordination movements.

At least 2 days before the experiment began, participants were led to the local school gym to undertake two standardized motor assessment tests that were used to determine the starting level of each participant’s coordinative abilities. We also administered a cognitive test to examine the students’ visuospatial abilities, attentive capacities and memory skills. The students were divided into two groups, and their individual testing took 30 min for each participant. The same tests were performed 2 days after the end of the training period.

All study procedures were performed within a school gym. Initial and final examinations of physical fitness and, cognitive testing, and all components of the exercise programs were administered during weekday mornings, at the same time of the day and under the same experimental conditions. We explained and demonstrated each task to the participants before they began each participant was given verbal encouragement and support throughout the process. If the participants made a procedural error during testing, we repeated, instructions and demonstrations, and the participant made a new attempt. Participants ingested no stimulating food or soft drink before testing, but otherwise maintained their normal food intake. The participants wore clothing and sport shoes appropriate for physical activity throughout the testing. The same physical education teacher supervised the exercise program and all measurements for testing, using a standardized test protocol. After the intervention, the physical education teacher was also asked to give a judgment of the intervention program and adolescent’s behavior.

### Measures

#### Slalom Bask Test

The first motor test was the “Slalom Bask Test” (Donati et al., 1994), used to measure motion spatial planning and coordinative abilities. This test assessed total body movement and, required participants to palm a basketball and dribble around a set obstacle course as quickly as possible (Dajic et al., 2017). It started with a 5 min warm-up period, after which participants were instructed about the exercise to be performed. Both verbal instructions and demonstrations were provided using a standardized protocol and included verbal encouragements throughout all tests to ensure maximal effort. Every test started with a 5 min habituation period during which participants were familiarized with the test. To perform the test, a starting line and an ending line were drawn on the floor at a distance of 20 m, and six cones were put on the same path, spaced 3 m from each other with the first cone spaced 3 m from the starting line. The participant began behind the starting line and went toward it, slaloming around cones and palming the basketball with one hand. If the participant lost the ball, it had to be recovered, and the exercise resumed from where it had been interrupted. Participants had to turn at the end, sprint back, and repeat the same procedure once. The main outcome measure was manually recorded time, taken with a hand-held stopwatch, with the best trial recorded for further data analysis. Reliability of this test was good (Intraclass coefficients or ICC: pre = 0.85; post = 0.88).

#### Throwing and Catching Test

The second motor test, called “Throwing and Catching Test” (Buonaccorsi, 2001), began after all participants finished the first test, with a 5 min rest interval between the two. The object of this test was to monitor the student’s abilities to coordinate visual information and then received control, guide, and direct the hands to catch a ball (hand-eye coordination) (Dirksen et al., 2016). At the end of a short warm-up, participants were instructed about how to perform the exercise. After a demonstration in which participants were instructed to throw and catch the rhythmic ball as fast as possible, they carried out a short test phase of five throws and catches immediately before the test started. For the test, one line was drawn 3 m from the wall and another was drawn on the wall at a height of 1.5 meters (strictly indicative because the throw could be done at any height). The participants stood behind the line on the floor and threw the ball against the wall trying to catch it before it fell to the floor. The throw was valid if the ball did not touch the ground and the participant remained behind the line. The test ended up when the participant completed 10 valid throws and catches. The time taken to accomplish each trial was taken with a hand-held stopwatch and recorded, and the best trial was used in further analyses. This test showed good reliability (ICC: pre-test = 0.89; post = test = 0.93).

#### Corsi’s Block-Tapping Test

We conducted a cognitive assessment with the Corsi Block-tapping Test, a valid neurocognitive test for visuospatial attention and visual working memory assessment (Kessels et al., 2000). It consisted of a series of nine identical blocks arranged irregularly with the sides of the cubes facing the examiner and numbered 1–9. The examiner tapped the block in a randomized sequence of increasing length, starting with two blocks. Immediately after each tapped sequence, the participant attempted to reproduce the sequence of taps, with the highest successfully recalled sequence called the Corsi span. The test began with a sequence of two units. The examiner presented three sequences for each series. If the participant exactly reproduced two of three sequences, a new increasingly long sequence was presented. Each time a maximum of five equal-unit sequences was tapped out (three from the examiner and two from the participant). The test ended when the participant obtained three incorrectsequences of the same length. Corsi’s test score was the longest number of items correctly reproduced at least twice, and this score represented the participant’s spatial memory span. Total testing time per participant ranged from 10 to 15 min, including the instructional and practice phase. The reliability of the test was between *r* = 0.81 and *r* = 0.89. Assessment protocol has been proposed before the beginning of the phase of observation and at the end of the intervention, to analyze any Corsi’s test score changes.

#### Exercise Training Intervention

The exercise training program was described to each participant before starting. Both the experimental and control groups undertook the supervised exercise sessions in a school gym twice weekly for 12 weeks, with the duration in each session approximately 60 min. The entire intervention program was implemented in 24 training sessions, with each session aimed at keeping the students’ effort at a medium-high level and to achieve a high volume, intensity and density of exercise. The rest period was very short for low-intensity activities and was 2–3 min for activities of medium to high intensity. As noted, a single physical education teacher supervised and performed the training exercises program with the collaboration of an experienced instructor who was a graduate in physical education training. To encourage learning and achieving the study’s objective, the teacher created a fun and active learning environment, using appropriate teaching styles and strategies (Ayers and Sariscsany, 2010).

Each training session started with a brief full-body dynamic warm-up and ended with cool-down exercises, for both groups. Warm-up included marching in place, wide toe touch, leg swings, arm swings, shoulder rotations, hip rotations, push-ups, lunges, walking jacks, jumping jacks, hip circles and bodyweight squats.

The experimental group received coordinative abilities training, following a specific exercise protocol designed to improve cognitive skills, including:

– Slalom circuits
– Dexterity circuits
– Jump rope exercises
– Throwing and catching exercises
– Static and dynamic balance exercises
– Jumps and direction changes exercises
– Rhythm exercises
– Hand-eye coordination and foot-eye coordination activities
– Motor responses exercises
– Motor differentiation exercises

The control group received a group training program for general psychophysical wellness, following a protocol with these features:

– Bodyweight exercises
– Group exercises with small training gear
– Joint mobility exercises
– Calisthenics basic workout
– Pilates exercises

Following these different exercise programs participants ended their training with a cool-down program consisting of a 5 min period of various static stretching exercises, including glute stretch, standing quad stretch, side bench stretches, arm-cross shoulder stretch, overhead triceps stretch, lower back stretch, abdominal stretch and child’s pose. This cool-down period was important for muscles to relax and for the improvement of joint range of motion.

### Statistical Analysis

We carried out statistical analyses using SAS JMP^®^ Statistics (Version < 14.3 >, SAS Institute Inc., Cary, NC, United States, 2018). We presented data as group mean (*M*) values and standard deviations (*SD*) and checked for assumptions of normality (i.e., Shapiro-Wilk test) and homogeneity of variances (i.e., Levene test) in the data distributions. We used an independent sample *t*-test to evaluate group differences at baseline and a two-way ANOVA (group (experimental/control) × time (pre/post-intervention), with repeated measures on the time dimension, was conducted to examine the effect of the Multilateral Training on all dependent variables. When “Group × Time” interactions reached significance, group-specific *post hoc* tests (i.e., paired *t*-tests) were conducted to identify the significant comparisons. Partial eta squared *(*η^2^_*p*_*)* was used to estimate the magnitude of the significant “Time × Group” interaction and interpreted using the following criteria: small (η^2^*_*p*_* < 0.06), medium (0.06 ≤ η^2^*_*p*_* < 0.14), large (η^2^*_*p*_* ≥ 0.14). Effect sizes for the pairwise comparisons were determined by Cohen’s *d* and interpreted as small (0.20 ≤ *d* < 0.50), moderate (0.50 ≤ *d* < 0.79) and large (*d* ≥ 0.80) (Cohen, 1992). Statistical significance was set at *p* < 0.05.

## Results

The physical education teacher gave a positive opinion on the feasibility, repeatability and utility of the intervention program, and he observed enthusiasm in participation, an improvement in relational dynamics (e.g., increased socialization and less aggressive behavior) and a growing interest in physical activity from the participants.

All participants received the treatment conditions as allocated and their average adherence (attendance) to intervention sessions was 89.6% (21.5 of 24 actual sessions). No injuries were associated with either training program. The groups did not differ significantly at baseline on anthropometric characteristics and they showed no meaningful difference at pre-test on the motor tests and the Corsi Block Tapping test (*p* > 0.05). Pre- and post-intervention results for all dependent variables are presented in Table 1.

**TABLE 1:** Changes after 12-week coordinative abilities training intervention.

	Experimental group (*n* = 30)			Control group (*n* = 30)		
	Baseline	Post-test	Δ	Baseline	Post-test	Δ
**Coordinative abilities**						
Slalom bask test (s)	15.90 (5.33)	14.40 (6.21)	−1.49(4.59)	18.27 (5.59)	17.29 (6.00)	0.97 (3.58)
Throwing and catching test (s)	21.40 (6.91)	19.32(5.70)†*	−2.08(2.49)	23.04 (6.56)	22.77 (6.73)	−0.27(1.16)
**Cognitive skills**						
Corsi’s block-tapping test (*n*)	3.93 (1.11)	5.53(1.59)†*	1.60 (1.27)	4.33 (1.37)	4.70 (1.47)	0.36 (1.12)

Values are presented as mean (± SD); Δ: pre- to post-training changes; ^†^Significant “Group × Time” interaction: significant effect of the intervention (p < 0.001). *Significantly different from pre-test (p < 0.001).

### Motor Ability Tests

A two-factor repeated measures ANOVA found a significant “Time × Group” interaction for the Throwing and Catching Test [*F*_(1, 58)_ = 12.97, *p* < 0.001, η^2^*_*p*_* = 0.18, large effect size]. *Post hoc* analysis revealed that the experimental group made significant improvements in the motor abilities performance (*t* = −4.57, *p* < 0.001, *d* = 0.83, large effect size), whereas no significant changes were found for the control group. Conversely, statistical analysis showed no significant “Time × Group” interaction for Slalom Bask Test [*F*_(1, 58)_ = 240, *p* = 0.626, η^2^*_*p*_* = 0.04, small effect size].

### Corsi’s Block-Tapping Test

Statistical analysis revealed significant “Time × Group” interaction for the Corsi’s block-tapping Test [*F*_(1, 58)_ = 15.72, *p* < 0.001, η^2^*_*p*_* = 0.21, large effect size]. The *post hoc* analysis revealed a significant improvements in the score for this cognitive skill (*t* = 6.87, *p* < 0.001, *d* = 1.25, large effect size) in the intervention group. No significant changes were found for the control group (*p* > 0.05).

## Discussion

This research aimed to investigate the relationship between physical fitness and cognitive skills, in particular, the effect of a 12-week coordinative exercise intervention program on visuospatial abilities (such as hand-eye coordination and motion spatial planning), attention performance and mnemonic skills in a school setting. Specifically, hand-eye coordination is a complex cognitive ability, as it calls for individuals to unite their visual and motor skills, allowing for the hand to be guided by the visual stimulation their eyes receive (Çetin et al., 2018). Instead, spatial planning allows to direct movement and help the brain understand where the body is located in space (self-perception) (Clark et al., 1994). They are especially important for the writing, and consequently for academic success.

Visuospatial attention is the process by which individuals select stimuli in their environment for perception and action (Smith and Chatterjee, 2008). It is important for reading and school subjects such as math, geometry, and drawing. Lastly, working memory, the ability to maintain and consciously manipulate information, helps kids hold on to information long enough to use it. Working memory plays an important role in concentration and in following instructions (Baddeley et al., 1999). Working memory is responsible for many of the skills children use to learn to read and learning math (Hahn and Buttaccio, 2017).

In this study, two groups of adolescent students performed a coordinative exercises intervention or standard physical education program and were assessed by two standardized motor tests and a cognitive test. The results revealed that a 12-week of coordinative exercise intervention program significantly improved cognition. This supports the idea that the coordinative character of the exercise is responsible for the significant difference between the two groups.

The first important finding of the present study concerns the positive impact that coordinative exercise appears to have on hand-eye coordination. This was confirmed by the fact that the group who practiced coordinative exercise showed the better ability of the vision system to coordinate the information received through the eyes to control, guide and direct movement, than the control group. This finding reveals the presence of neurocognitive effects of the training on the visuospatial task (Szabo et al., 2020).

In contrast to the positive effects founded on hand-eye coordination, another important finding of our research concerns the null impact that coordinative exercise appeared to have on the improving motion spatial planning compared to the control group. This was confirmed by the fact that, compared to the control group, the experimental group showed the same results in completing its motor tasks. This result seems to be in conflicts with the nature of the coordinative exercise. Most likely, the acquisition and retention of the coordinative tasks, assessed through the Slalom Bask test, require a longer period of training. Therefore, it would be appropriate to organize more complex forms of exercise, such us multilateral training (Fischetti and Greco, 2017; Latino et al., 2019). In this way, the consolidation process of the spatial planning might require a longer lead time than attention span and mnemonic abilities, which are more sensitive to exercise. Future studies will be needed to compare, extending the duration of related work, the time of acquisition, retention, stabilization and decay of the motion spatial planning.

Perhaps the most striking finding of the present study was the capacity of the coordinative exercise program to improved visuospatial attention and visuospatial working memory. This result was a consequence of the fact that the experimental group manifested a better performance in storing, focusing attention on, and manipulating information for a relatively long period of time. This type of exercise appears to improve cognitive learning through optimization of visual and spatial working memory (Latino et al., 2020). These changes allow the selection of a more appropriate cognitive control strategy (Ludyga et al., 2018). Effective learning depends above all on working memory as new information is continuously built upon previous knowledge as a student progresses. Being able to hold information in mind and recall previous learnings while engaged in school tasks is essential for students to develop academically (Allen et al., 2019). Moreover, the experimental group showed a better performance in the ability of select stimuli to direct and orient the action. In fact, spatial attention allows to selectively process visual information through prioritization of an area within the visual field (Voinea et al., 2019).

In connection with what was found in this study, the findings are in line with previous studies displaying correlations between coordinative exercise and cognition (Planinsec, 2002; Uhrich and Swalm, 2007; Budde et al., 2008; Yu-Kai et al., 2013). Several studies, which focused on the association between physical exercise and cognitive function during adolescence, have documented a positive relationship between physical fitness and academic achievement (Shephard et al., 1994; Shephard, 1997; Donnelly and Lambourne, 2011). Many other studies have demonstrated that coordination training elicits a cascade of neurological changes in the hippocampus linked to memory consolidation and skilled actions (Gomez-Pinilla and Hillman, 2013; Rehfeld et al., 2017). The coordinative exercise training program is capable of optimizing brain networks (Phillips et al., 2014) involved in working memory, attention span and visuospatial coordination (Voelcker-Rehage et al., 2011; Hötting and Röder, 2013; Niemann et al., 2016). It is also capable of creating a reserve of precursor cells that influence individuals’ learning capabilities throughout the whole of their life span (Kopp, 2012). Additionally, coordinative exercise with both low and moderate intensities could increase the allocation of attentional resources and shorten the time needed for neurocognitive processing (Yu-Kai et al., 2013). Rogge et al. (2017) suggested that a combination of coordination exercise and balance training improved memory and spatial cognition among healthy adults (Dunsky, 2019). Furthermore, coordinative abilities training was associated with high activation in visual-spatial networks in the brain of older adults (Crush and Loprinzi, 2017). Therefore, based on what was discussed, it is possible to claim that the behavioral results of the previous research support our hypothesis, in which students had significantly improved cognitive function following the coordinative exercise intervention.

Despite the contribution regarding the relationship of physical fitness to academic achievement, some limitations are to be considered in this study. First, the small sample size due to the difficulties in recruiting motivated students to participate. Second, the sample was recruited from a population of students at a single, large, public school, thus, the results may not be generalizable to students at other institutions or with other demographics. A third limitation was that the effect of exercise on mood and fatigue, which are both sensitive to acute exercise and could impact memory and attention, was not assessed. Future research would need to examine these issues to explain these variables. However, the results obtained could provide important indications for future studies. The strength of this study was that our results extend the knowledge base on coordinative exercise and add new evidence indicating that the benefits of this intervention on memory, attention and visuospatial span consolidation might generalize to other forms of cognitive skills.

## Conclusion

In conclusion, our results suggest that coordinative abilities training intervention can be used to enhance cognitive skills through an optimization of the consolidation of coordinative abilities. This positive effect of coordinative exercise has significant perspectives in both sports and school achievement. These findings could inform teachers that coordinative exercise, carried out twice per week, in 60 min sessions for 12 weeks might serve as a useful intervention to improve the academic performances of adolescent students. More studies will be required to determine if these promising results will create new perspectives in the search. Only then, we will be able to be accurate in the prescription of personalized coordinative exercise interventions to optimize cognitive abilities.

Therefore, we believe that further research is necessary to better determine the role that physical fitness has on academic achievement. Most importantly, it would be to know the potential influence of a coordinative ability training program on the brain, in particular, on the increased synthesis and expression of BDNF (Brain-Derived Neurotrophic Factor). Specifically, it should be examined the relationship between this neurotrophin and both fitness and cognitive performance. Physical exercise would seem to lead a significant increase of BDNF expression (Jonasson et al., 2017), which can produce an improvement of visual, physical, and cognitive stimulation that leads to more neuronal activity and synaptic communication (Håkansson et al., 2017). Additionally, future research should progressively reach higher levels of challenges, by finding more complex forms of exercise involving both motor and cognitive tasks.

## Data Availability Statement

The raw data supporting the conclusions of this article will be made available by the authors, without undue reservation, to any qualified researcher.

## Ethics Statement

The studies involving human participants were reviewed and approved by the University of Aldo Moro. Written informed consent to participate in this study was provided by the participants’ legal guardian/next of kin.

## Author Contributions

FL designed the study, conducted the research, collected data, carried out the statistical analysis, was involved in the interpretation of data, and wrote and revised the manuscript. SC collected data and was involved in the interpretation of data and revised the manuscript. FF coordinated the study and interpreted the data and revised the manuscript. All authors contributed intellectually to the manuscript, and read the manuscript and approved the submission.

## Conflict of Interest

The authors declare that the research was conducted in the absence of any commercial or financial relationships that could be construed as a potential conflict of interest.
